# Precambrian origins of the TNFR superfamily

**DOI:** 10.1038/cddiscovery.2016.58

**Published:** 2016-07-18

**Authors:** S D Quistad, N Traylor-Knowles

**Affiliations:** 1 Biology Department, San Diego State University, San Diego, CA, USA; 2 Laboratoire de Génétique de l’Evolution (LGE), Institute of Chemistry, Biology, and Innovation, ESPCI ParisTech/CNRS UMR 8231/PSL Research University, Paris, France; 3 Laboratoire de Colloïdes et Matériaux Divisés (LCMD), Institute of Chemistry, Biology, and Innovation, ESPCI ParisTech/CNRS UMR 8231/PSL Research University, Paris, France; 4 The Rosenstiel School of Marine and Atmospheric Sciences, Marine Biology and Ecology, University of Miami, 4600 Rickenbacker Causeway, Miami, FL 33149, USA

## Abstract

The evolution of the tumor necrosis factor/tumor necrosis factor receptor superfamily (TNF/TNFR) is complicated and not well understood. To date, most TNFR studies have focused on vertebrate models leaving the role of TNFRs in invertebrates largely unexplored. The evolution of important cellular processes including stress response, apoptosis, development, and inflammation will be better understood by examining the TNF/TNFR superfamily in ancient invertebrate phyla. How widespread is this gene family within the evolutionary tree of life and is there evidence for similar function in invertebrates? A first step is to identify the presence or absence of these genes within basal metazoan taxa using the signature cysteine-rich domain (CRD) of the TNFR superfamily. In this perspective, we will start by examining what is currently known about the function of TNFRs in invertebrates. Then, we will assess the role of TNFRs in apoptosis and explore the origins of the domains found in TNFRs including the death domain (DD) and CRD. Finally, we will examine the phylogenetic relationship between TNFRs containing DDs identified to date. From these data, we propose a model for a Precambrian origin of TNFRs and their functional role in apoptosis.

## Facts

- The TNFR superfamily is remarkably diverse across animal phyla.
- The role of TNFRs in apoptosis is ancient.
- The cysteine-rich domain (CRD) of TNFRs is present across all domains of life.

## Open questions

- Why is the TNFR repertoire of choanoflagelletes so diverse?
- What is of the functional role of invertebrate TNFRs that lack death domains?
- When did the TNF-ligand and its role in apoptosis emerge?
- What are the ligands of invertebrate TNFRs?

## A Brief History of TNF/TNFR Research

Although the focus of this study will be on the function and origin of invertebrate TNFRs, a brief history of the TNF/TNFR superfamily is required to place these studies in proper context. The path towards discovery can be traced back to the late nineteenth century when the regression of human tumors was observed following bacterial infection.^1^ These results led to the treatment of human cancers using bacterial extracts termed ‘Coley’s toxins’, named after the oncologist W. Coley.^2^ More than 50 years later, lipopolysaccharide was isolated from these extracts and shown to induce tumor regression; however, it was determined that it was a factor in the serum, termed tumor-necrotizing factor that actually caused the tumor regression. The cellular receptors for TNF were first described in 1984 leading to the discovery of a novel protein domain termed death domain (DD) that was located on the intracellular portion of TNFRs and was responsible for initiating programmed cell death (apoptosis).^3–5^ These ‘Death Receptors’ were shown to be central regulators of apoptosis serving as both internal and external sensors that maintain organismal integrity.^5^ In humans, a total of 19 different TNF ligands and 29 TNFRs have been described to date.^6^ In addition to apoptosis, the TNF/TNFR superfamily is involved in cellular differentiation and proliferation through the activation of a variety of signaling pathways. For a more detailed description of these pathways and the many cellular processes that the TNF/TNFR superfamily is involved in, the authors point the reader to the review by Gaur and Aggarwal.^7^ TNFRs have also been extensively studied in other vertebrate model systems such as zebrafish^8^ and mice,^9^ however the focus of this study will be exclusively on invertebrate TNFRs.

## Chordata

The phyla Chordata is defined by the presence of a notochord during development and includes both invertebrate and vertebrate species. Invertebrate members of the phyla chordata (e.g., tunicates, hagfish, and cephalochordates) are considered to be the most closely related to humans, and have been the focus of many studies on diversity of the innate immune system and its divergence from the adaptive immune system.^10^ For example, in the tunicates, *Ciona savignyi* and *Ciona intestinalis,* TNF-*α* was upregulated in hemocytes in response to a lipopolysaccharide challenge and was primarily expressed in inflammatory granulocytes that occurred at the area of inflammation.^11^ The lancelet fish, *Amphioxus,* has an extensive repertoire of TNF/TNFRs present in its genome.^12^ In response to an lipopolysaccharide immune challenge, moderate upregulation of TNF and TNFR occurred and it was a dynamic response depending on the homolog. From these data, it was concluded that TNF and TNFR could be important in the immune response to mucosal pathogens in *Amphioxus.*^13^ Overall, the TNFR superfamily has been demonstrated to have a dynamic role in immune function and is activated in response to immune challenges from lipopolysaccharide in multiple chordate species.^11–13^

## Mollusca

Members of the TNF/TNFR superfamily have been widely described in the phylum Mollusca (e.g., oysters, mussels, and clams) and most of the evidence points to its role in the immune response to Gram-negative bacteria. For example, the first TNF-*α* homolog was characterized in Disk Abalone (*Haliotis discus discus*) and was constitutively expressed in infected and non-infected tissues. However, it was significantly upregulated in the gills that were exposed to multiple pathogens.^14^

In the Pacific Oyster (*Crassostrea gigas*), TNFRs has an important role in biotic and abiotic stress response, specifically six of the Pacific Oyster TNFRs were differentially expressed in response to bacteria exposure.^15^ In addition, many of the Pacific Oyster TNF ligands exhibit a dynamic gene expression response pattern, where more than half of the TNFs are responsive to biotic challenges. Pacific Oysters infected with *Vibrio sp.* also had several different TNFs that responded differently; four TNFs are late responders, three are repressed, and one is a very early responder.^15^ Although the complete picture of the mechanisms controlling this TNF expression is not completely understood, it is apparent that the expression is sensitive to timing during an infection, and is dynamic in what does and does not respond.

In the Flat Oyster (*Ostrea edulis*), TNF expression was upregulated in hemolymph cells during a *Bonamiosis* infection.^16^ This upregulation appears to be correlated with the severity of the infection, and again presents the nature of TNF/TNFR expression as dynamic.^16^ Finally, in the scallop (*Chlamys farreri*), TNFR was upregulated in the gill tissues and mantle in response to a *Listonella anguillarum* challenge.^17^

Overall, the TNF/TNFR system in mollusks is important in the host response to bacterial infection. The TNF/TNFR system responds to challenges with different Gram-negative bacteria, and appears to have a dynamic nature, which changes depending on severity, timing, and tissue that is infected.^14–17^

## Arthropoda

Information on the role of the TNF/TNFR signaling system in arthropods is limited to the fruit fly (*Drosophila melanogastor*), which has a TNF homolog named Eiger and a TNFR homolog called Wegen.^18^ Although there only appears to be one TNF and one TNFR in *Drosophila*, the work to date has been the most extensive on any invertebrate. The role of the Eiger/Wegen system in immunity is very intricate, where tolerance phenotypes for intracellular Gram-positive bacteria have been observed, and faster death is observed in Eiger mutants exposed to extracellular microbes.^19^ In addition, Eiger mutants in *Drosophila* appear to affect many components of the immune system including melanization, Imd signaling, and the response to genotoxic stress.^20^ Finally, Eiger signaling has other important functions in the sensory system of *Drosophila* such as its involvement in the sensitization of nociceptive sensory neurons during thermal allodynia and UV exposure.^21^

Similar to Mollusca, the TNF/TNFR in *Drosophila* and presumably in other arthropods is involved in response to pathogens, but has a very complicated response. In addition, the role of TNFR in the neurosensory system of *Drosophila* adds in another layer of complexity to the function of this gene family.

## Cnidaria

Cnidarians, which include corals, sea anemones, jellyfish, and hydrozoans, were previously thought to be ‘simple’ organisms that would also possess correspondingly simple immune systems. However, recent evidence has demonstrated that despite their morphological simplicity, the innate immune system of cnidarians is complicated and diverse.^22–24^ This complexity and diversity is particularly apparent in the TNF/TNFR superfamily.^25^

In corals, the activation of the TNF/TNFR system has been demonstrated in response to heat stress.^26,27^ In the coral *Acropora hyacinthus*, heat-tolerant colonies express different TNFRs compared with heat-sensitive corals suggesting they may be important in the adaptation of corals to future climate change.^27^ In Hydra, a TNFR protein was expressed at the tips of the wound site suggesting it could be involved in signal transduction during wound healing.^28^ To date, no publications examining the role of the TNF/TNFR superfamily in response to pathogenic insult have been reported in Cnidarians, however, the conserved role of TNFRs in the bacterial immune response observed in Arthropods, Mollusks, and Chordates suggests that TNFRs may also be involved in Cnidarian immunity.

## TNFRs and Apoptosis

Whereas most invertebrate TNFR studies have focused on the role of TNFRs in physiological stress and immunity, recent work demonstrated the TNF-apoptotic response is evolutionarily conserved in the coral species *Acropora yongei*. A recombinant human TNF-*α* was shown to bind to coral cells in culture, increase caspase activity, and finally cause cell death. The reciprocal experiment, exposure of a purified coral TNF to human cells, also induced cell death through a TNFR signaling pathway suggesting over 550 million of evolutionary conservation between humans and corals.^25^ Whereas it has been demonstrated that TNF-induced apoptosis is conserved in corals, additional proteins involved in the apoptotic cascade are also present.^29,30^ Functional studies have demonstrated that the Caspase-8 of *A. millepora* also displays the same substrate specificity as human Caspase-8 and interacts directly with FADD. In addition, the expression of coral Bcl-2 family members in mammalian cells demonstrated functional conservation of the anti-apoptotic/effector responses.^31^ Although direct experimentation with Cnidarian apoptotic proteins is still in its infancy, multiple lines of evidence suggest the machinery of TNFR-mediated apoptosis was present in the last common metazoan ancestor.^30^ To better understand the observed conservation of TNFR-mediated apoptosis in corals, it is necessary to investigate the domains of the TNFR protein itself, which domains link a specific TNFR to the apoptotic response, and where these domains are found throughout the tree of life.

### The functional domains of TNFRs

Despite remarkably low sequence similarity, all members of the TNFR superfamily possess at least one extracellular cysteine-rich domain (CRD) consisting of six cysteines that form three disulfide bridges.^32^ The intracellular portion of individual TNFRs is variable and can be attributed to the variety of biological activities they are involved in including immune modulation, inflammation, and cell differentiation.^33^ However, TNFRs that are involved in programmed cell death or apoptosis share an intracellular DD that activates the apoptotic cascade.^34^

### Origins of CRDs, DDs, and TNFRs

**Figure 1:**
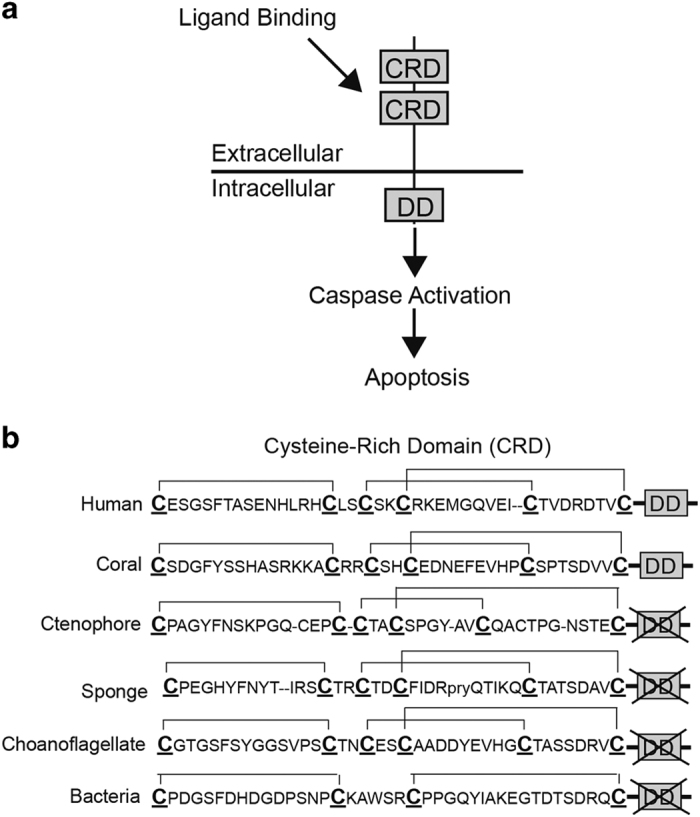
Evolution of TNFR domains across phyla. (**a**) TNFRs possess an extracellular CRD involved in ligand binding and an intracellular DD for the initiation of apoptosis. (**b**) The CRD across animal phyla and domains of life. The CRD of TNFRs is approximately 40 amino acids in length and contains three conserved disulfide bridges between C1-C2, C3-C5, and C4-C6.

**Figure 2:**
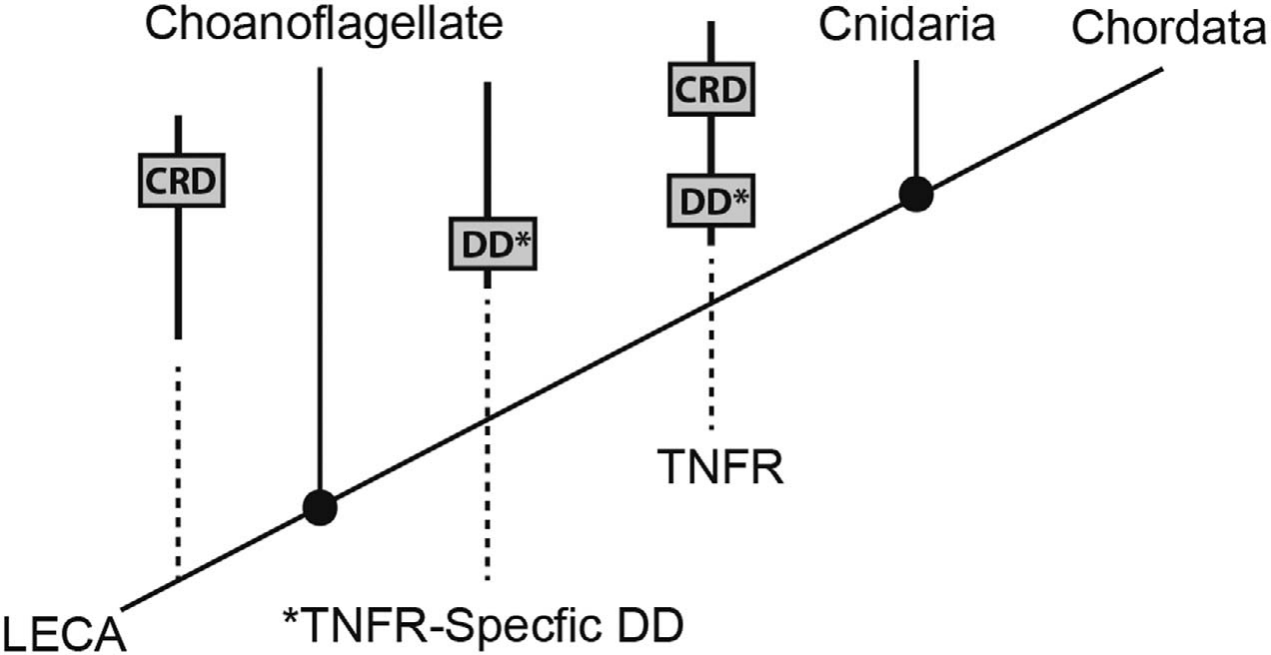
Hypothesis for the origin of TNFRs. The CRD existed in the last common eukaryotic ancestor (LECA) while the TNFR-specific DD emerged before the last common metazoan ancestor (LCMA). Following a fusion event between a CRD and DD, the primordial TNFR involved in apoptosis was formed.

The CRD has deep evolutionary origins and can be found within the predicted proteomes of Cnidarians,^29,35,36^ Choanoflagellates,^37^ sponges,^38^ Ctenophores,^39,40^ bacteria (PRJNA177747), and plants^41^ (Supplementary Table S1). To date, the Choanoflagelletes (*Monosiga brevicollis* and *Salpingoeca rosetta*) have the highest number of CRD-containing proteins with 44 and 84, respectively (Table 1). However, none of these CRD-containing proteins or any CRD protein found within the repertoires of other Choanoflagelletes, sponges, Ctenenophores, bacteria, or plants also possess a DD suggesting these TNFRs are not involved in apoptosis (see Supplementary Table S1 for full domain analysis). On the basis of the phylogenetic distribution of DD proteins, the domain was likely present in the last common metazoan ancestor. An independent BLAST search for TNFR-associated DDs identified positive matches within the genomes of Ctenophores, sponges, and Placozoans,^42^ however, none of these proteins also possessed a CRD (Figure 1 and Supplementary Table S2). Taken together, we propose that the CRD emerged in the last common eukaryotic ancestor followed by a fusion event with a DD during the last common metazoan ancestor resulting in the first TNFR involved in apoptosis (Figure 2). Alternatively, the CRD and DD could have independently fused into a single protein in multiple lineages. We conclude that the former hypothesis, a single fusion event as the most parsimonious explanation of the evolutionary conservation of TNF-induced apoptosis.^25^

### Discussion of phylogenetic tree

**Figure 3:**
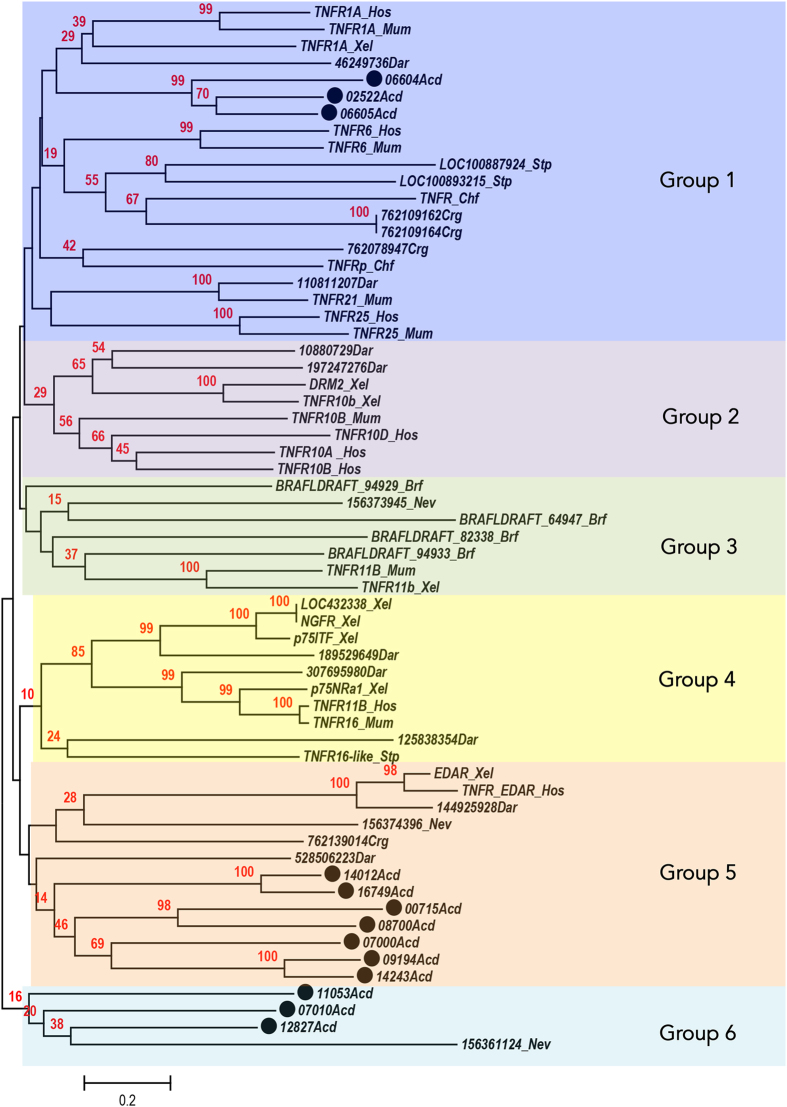
Evolutionary relationships of taxa with DD-containing TNFRs. The evolutionary history was inferred using the neighbor-Joining method.^44^ The optimal tree with the sum of branch length=30.30949398 is shown. The percentage of replicate trees in which the associated taxa clustered together in the bootstrap test (1000 replicates) are shown above the branches.^52^ The tree is drawn to scale, with branch lengths in the same units as those of the evolutionary distances used to infer the phylogenetic tree. The evolutionary distances were computed using the Poisson correction method^53^ and are in the units of the number of amino acid substitutions per site. The analysis involved 62 amino acid sequences. All positions containing gaps and missing data were eliminated. There were a total of 102 positions in the final dataset. Evolutionary analyses were conducted in MEGA7.^54,55^ Abbreviations of taxa include: *Homo sapiens (Hos), Mus musculus (Mum), Danio rerio (Dar), Xenopus laevis (Xel), Branchiostoma floridae (Brf), Strongylocentrotus purpuratus (Stp), Crassostrea gigas (Crg), Chlamys farreri (Chf), Nematostella vectensis (Nev),* and *Acropora digitifera (Acd)* with a black circle next to taxon name. For full description of sequences, see Supplementary Table 1.

To infer phylogenetic relationships between DD containing TNFRs neighbor-joining analysis was preformed on 62 amino acid sequences of the TNFR protein of representative vertebrates and invertebrates (Figure 3). Our neighbor-joining tree confirms that the sequence diversity previously found in vertebrate TNFRs is also present in invertebrates.^43,44^ Invertebrate TNFRs containing DDs have incredible variation, both within taxa and throughout the evolutionary tree. In our neighbor-joining analysis, we identified six clusters of TNFRs. The *A. digitifera* proteins are present within three groups (Group 1, Group 5, and Group 6). Within Group 1, *A. digitifera* proteins 06604Acd, 02522Acd, and 06605Acd are clustered together with mammalian TNFR1A. Although this TNFR is involved in apoptosis, its main function is in the regulation of pro-inflammatory signaling pathways.^45^ In Group 5, 7 of the 13 coral TNFRs cluster with the TNFR EDAR proteins. These proteins have previously been shown to be important for epidermal morphogenesis.^46^ Further testing would need to be done in corals to understand whether this is occurring, but it is possible that this role in morphogenesis could be a very primitive role for this extensive gene family.

Taken together, the DD-associated TNFRs from corals cluster into groups that contain TNFR members functioning in inflammation, morphogenesis, and apoptosis. The inflammation response of TNFRs has been widely studied in other invertebrates, but warrants more study in basal invertebrates (including corals, sponges, ctenophores, and so on). Although not yet functionally confirmed, this same function could exist in corals, and could therefore represent the most ancestral function of DD TNFRs. This would expand the functional repertoire of ancestral DD TNFRs to include not only apoptosis, but also inflammation and morphogenesis.

## Future Work

To investigate these hypotheses, the DD-binding partners of TNFRs should be determined. If a single fusion event occurred in the basal metazoan ancestor resulting in the fixation of TNFR-induced apoptosis, we would expect similar proteins to associate with the DD across phyla. In corals, a key unknown is the localization of specific TNFRs within coral tissue. Characterizing which cell types express TNFRs containing DDs will provide insight into what the ancestral state of TNFR-mediated apoptosis may have been. Whereas corals currently lack robust molecular tools for mechanistic investigations, two fellow Cnidarian model systems, *Nematostella vectensis* (sea anemone) and *Hydra magnipapillata*, have well-developed methods to investigate protein function.^47,48^ Apoptosis in general has been examined in *N. vectensis*^49,50^ and *H. magnipapillata,*^51^ but TNFRs have yet to be directly investigated. An integrative approach that involves multiple Cnidarian species combined with additional basal metazoan phyla is required to better understand the origin of evolution of TNFR-mediated apoptosis. Finally, although this review has shown that the function of apoptosis is likely a very old, there are other possible functional avenues to explore, including a role in inflammation and morphogenesis.

## Figures and Tables

**Table 1:** Summary of TNFRs across phyla

*Organism*	*Phyla*	*# TNFR (DD)*
*Homo sapiens*	Chordata	29 (8)
*Mus musculus*	Chordata	26 (7)
*Danio rerio*	Chordata	41 (9)
*Xenopus laevis*	Chordata	10 (9)
*Branchiostoma floridae*	Chordata	10 (4)
*Ciona intestinalis*	Chordata	3 (0)
*Strongylocentrotus purpuratus*	Echinodermata	13 (3)
*Azumapecten farreri*	Mollusca	2 (2)
*Crassostrea gigas*	Mollusca	17 (4)
*Drosophila melanogaster*	Arthropoda	1 (0)
*Daphnia pulex*	Arthropoda	1 (0)
*Acropora digitifera*	Cnidaria	40 (13)
*Nematostella vectensis*	Cnidaria	8 (3)
*Monosiga brevicollis*	Choanoflagellate	44 (0)
*Salpingoeca rosetta*	Choanoflagellate	84 (0)
*Mnemiopsis leidyi*	Ctenophora	13 (0)
*Pleurobrachia bachei*	Ctenophora	5 (0)
*Amphimedon queenslandica*	Porifera	2 (0)

Total number of proteins for each organism that contain a CRD with the number of those CRD-containing proteins also possessing a DD indicated in parentheses. See Supplementary Table 1 for full domain analysis.

## Abbreviations

TNF: tumor necrosis factor
TNFR: tumor necrosis factor receptor
CRD: cysteine-rich domain
DD: death domain
LPS: lipopolysaccharide
