# Supplementary figures and images for: Precambrian origins of the TNFR superfamily
Source: Cell Death Discov. 2016 Jul 18;2:16058–. doi: 10.1038/cddiscovery.2016.58 (PMC4979521; doi:10.1038/cddiscovery.2016.58)

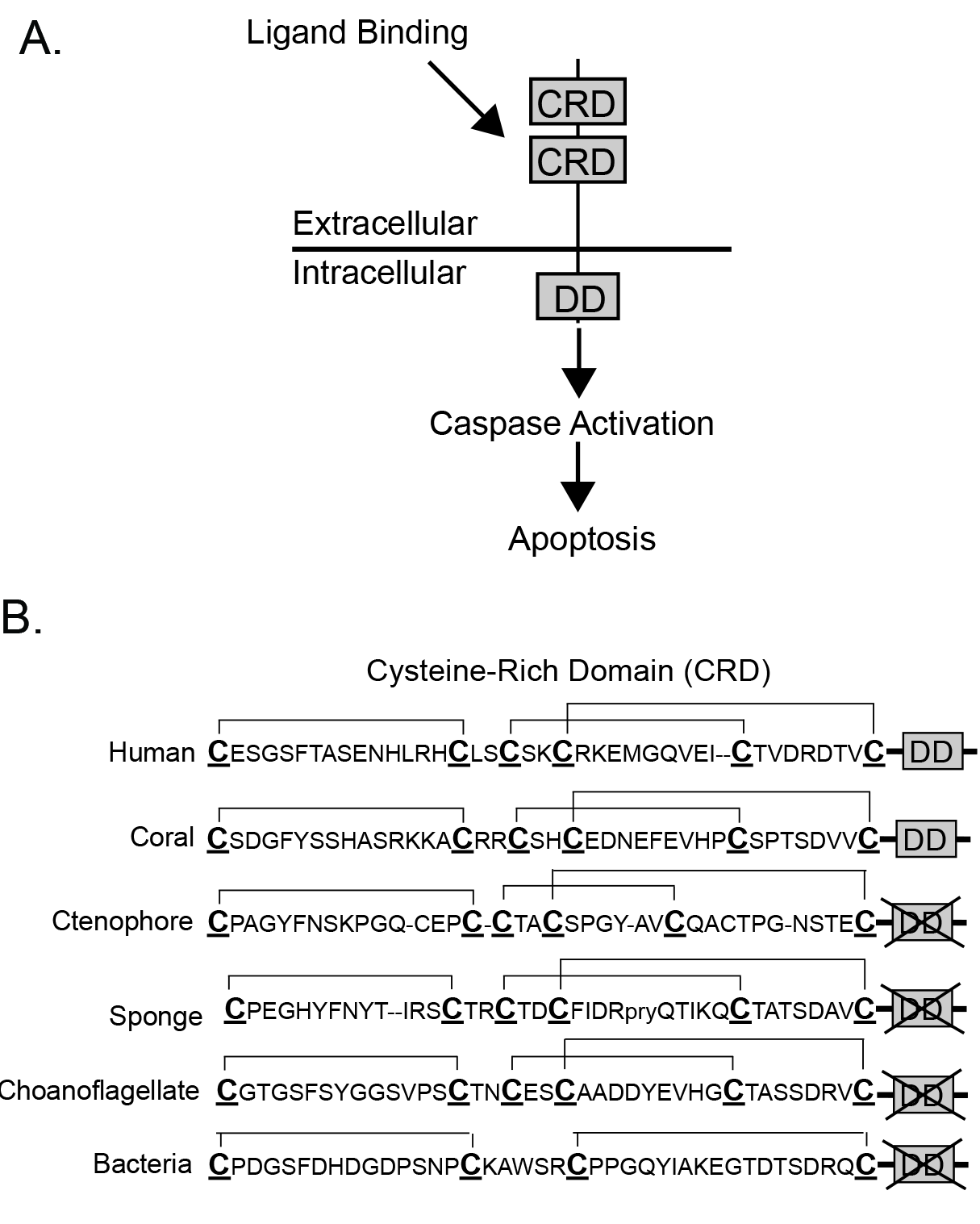

Supplement: Supplementary Figure 1 [file cddiscovery201658-s1.tiff]
